# Computer Simulation and Analysis on Flow Characteristics and Distribution Patterns of Polymethylmethacrylate in Lumbar Vertebral Body and Vertebral Pedicle

**DOI:** 10.1155/2015/160237

**Published:** 2015-12-07

**Authors:** Da Liu, Xu-li Liu, Bo Zhang, Dong-fa Liao, Zhi-qiang Li, Jiang-jun Zhou, Xia Kang, Wei Zheng, Wei Lei

**Affiliations:** ^1^Department of Orthopaedics, Chengdu Military General Hospital, No. 270, Rongdu Avenue, Jinniu District, Chengdu, Sichuan 610083, China; ^2^Department of Orthopaedics, Naval Hospital, No. 6, Mazhang District, Zhanjiang, Guangdong 524096, China; ^3^Department of Orthopaedics, Hospital of Nanjing Military Region, No. 4, Hudong Street, Yingtan, Jiangxi 335000, China; ^4^Department of Orthopaedics, Xijing Hospital, Fourth Military Medical University, No. 15, Changle West Road, Xi'an, Shaanxi 710032, China

## Abstract

This study was designed to analyze the flow and distribution of polymethylmethacrylate (PMMA) in vertebral body through computer simulation. Cadaveric lumbar vertebrae were scanned through electron beam tomography (EBT). The data was imported into Mimics software to build computational model. Vertebral body center and junction of pedicle and vertebral body were chosen as injection points. Silicone oil with viscosity of 100,000 cSt matching with PMMA bone cement was chosen for injection. The flow and distribution of silicone oil were analyzed using Fluent software. In vertebral body, silicone oil formed a circle-like shape centered by injection point on transverse and longitudinal sections, finally forming a sphere-like shape as a whole. Silicone oil diffused along lateral and posterior walls forming a circle-like shape on transverse section centered by injection point in pedicle, eventually forming a sphere-like shape as a whole. This study demonstrated that silicone oil flowed and diffused into a circle-like shape centered by injection point and finally formed a sphere-like shape as a whole in both vertebral body and pedicle. The flow and distribution of silicon oil in computational model could simulate PMMA distribution in vertebral body. It may provide theoretical evidence to reduce PMMA leakage risk during percutaneous vertebroplasty.

## 1. Introduction

Percutaneous vertebroplasty (PVP) is currently a common and effective treatment for vertebral lesions (e.g., high risk osteoporotic vertebrae, osteoporotic vertebral fractures, and vertebral tumor) [1–4]. However, bone cement leakage is one of the most common and serious complications [5–7]. Unclearness of the flow characteristics and distribution patterns of bone cement in the vertebral body is the key factor of this complication. For this reason, the related studies on the flow characteristics and distribution pattern of PMMA in the vertebral bodies were necessary to perform to provide theoretical basis to decrease the cement leakage risk in PVP [8–11].

In this study, the process of PMMA injection into vertebral body was simulated by computer program and the flow characteristics and distribution pattern of PMMA were further analyzed and measured using related software. We tend to explore the flow characteristics and distribution pattern of bone cement in vertebral body aiming at reducing the cement leakage risk during PVP.

## 2. Materials and Methods

### 2.1. Specimens

Vertebral specimens (L1–L4) were collected from four fresh cadavers (3 males and 1 female) aged from 24 to 38 years (average 31 years) provided by the Department of Anatomy of Fourth Military Medical University. All specimens were examined by X-ray analysis to exclude vertebral fracture, deformity, and osteolysis resulting from malignancy. Surrounding soft tissue was then removed and single lumbar vertebral bodies were separated from intervertebral discs. Vertebral bodies were degreased using 95% alcohol and stored in a freezer at −76°C for later use.

### 2.2. Computational Modeling

Continuous electron beam tomography (EBT) (GEImatronC-150EBCT, USA) scan was performed on the vertebrae (L1–L4) collected previously. Scanning parameters were as follows: slice thickness 2 mm, slice increment 2 mm, scan time 0.3 s, tube voltage 130 kV, and electric current 620 mA. The acquired EBT data were then imported into Mimics software (V10.01, Materialise, Belgium), and 3D reconstruction was performed after bone mineral density CT value was selected. The average value of vertebral shape was chosen to simulate computational models of vertebral body shape (appendix of vertebra excluded). Vertebral bodies were cut into pieces and one piece of every vertebra was observed by scanning electron microscope (SEM) to measure the poriness and average pore diameter. The poriness of vertebra samples in this study was measured to be about 70–80%, and the average poriness in computational model was 75% according to the related literatures. The average pore diameter was 0.90 ± 0.22 mm.

Silicone oil with viscosity of 100,000 cSt (Dow Corning, USA) matching with the PMMA was chosen for injection. The unsteady incompressible viscous laminar flow in the porous medium was used as a flow model to analyze the flow characteristics and distribution pattern of PMMA inside the vertebral bodies. Due to the flow characteristics and boundary conditions, Darcy Law $\left(\nabla P=-\left(\mu /\alpha \right)\vec{v}\right)$ was chosen as the constitutive equation in the transfer process of porous medium. Numerical simulation of the injecting and flowing of PMMA was performed by Fluent software (V6.3, ANSYS, USA), and the flow characteristics and distribution patterns of PMMA were analyzed using Euler's method.

### 2.3. Simulation and Analysis of Silicone Oil Distribution in Vertebral Body

**Figure 1:**
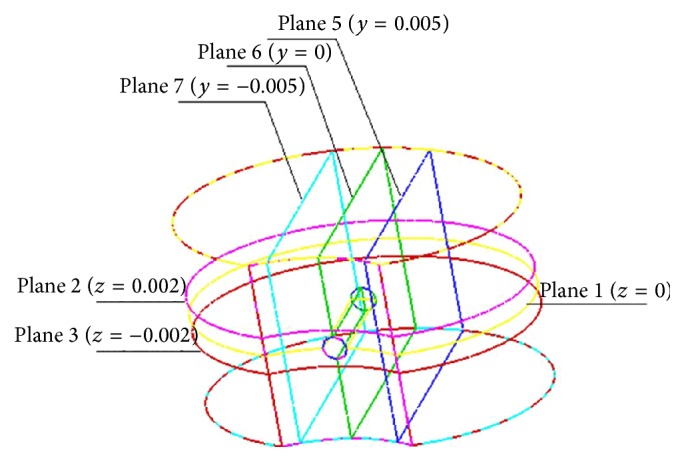
Spatial structure of a vertebral body and the observing sections.

The posterior wall and physical center of the vertebral model were selected as the insertion point and the injection point, respectively. Silicone oil with a viscosity of 100,000 cSt was injected at the physical center inside the vertebral body. As shown in Figure 1, the physical center of the vertebral body was also set as the origin of a 3D coordinate system with the long axis of the vertebral body as *y*-axes, short axis as *x*-axes, and vertical axis as *z*-axes. During the analysis, transverse section through the injection point was set as the datum plane (plane 1, *z* = 0) of the vertical axis, defined as positive above the datum plane and negative below the datum plane. Two additional symmetric transverse sections (plane 2, *z* = 0.002; plane 3, *z* = −0.002) were selected to simulate and analyze the flow characteristics and distribution patterns of silicon oil on the transverse sections in vertebral body. Longitudinal section through the injection point was set as the datum plane (plane 6, *y* = 0) of the long axis, defined as positive on the right and negative on the left. Similarly, two additional symmetric longitudinal sections (plane 7, *y* = −0.005; plane 5, *y* = 0.005) were selected to simulate and analyze the flow characteristics and distribution patterns of silicon oil on the longitudinal sections in vertebral body. Diameters of the distribution range on both transverse and longitudinal sections were calculated.

### 2.4. Simulation and Analysis of Silicone Oil Distribution in Pedicle

The junction of the pedicle and the vertebral body was selected as the injection point. Silicone oil with a viscosity of 100,000 cSt was injected at this point to simulate injection in pedicle. The injection point was set as the origin of a 3D coordinate system with the long axis of the vertebral body as *y*-axes, short axis as *x*-axes, and vertical axis as *z*-axes. During the analysis, transverse section through the injection point was set as the datum plane (plane 1, *z* = 0) of the vertical axis, defined as positive above the datum plane and negative below the datum plane. Two additional symmetric transverse sections (plane 2, *z* = 0.002; plane 3, *z* = −0.002) were selected to simulate and analyze the flow characteristics and distribution patterns of silicon oil on the transverse sections in vertebral pedicle. Diameters of the distribution range on transverse sections were also calculated. The transverse section (plane 4, *z* = 0.0097) 0.3 mm distant to the upper endplate was selected to simulate and analyze the flow and distribution of silicon oil when it reaches the endplate wall. Diameters of the transverse distribution range were also calculated. The ring-shaped sections of lateral and posterior walls of vertebral body around the injection point were selected to analyze the flow and distribution of silicon oil when it reaches lateral vertebral wall and posterior vertebral wall, respectively.

## 3. Results

### 3.1. Silicon Oil Distribution on Transverse Sections in Vertebral Body

**Figure 2:**
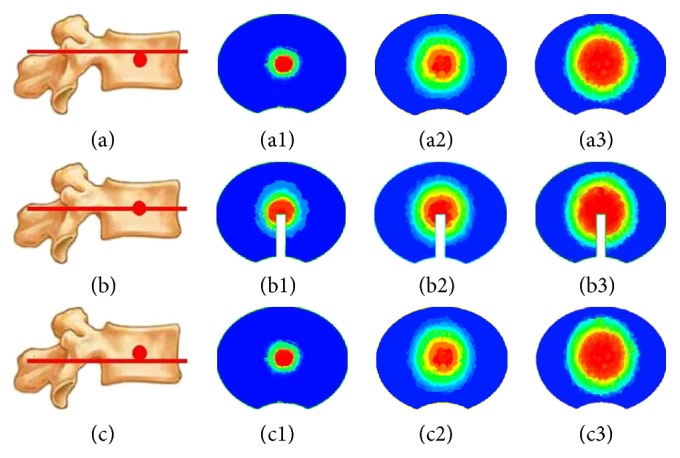
Simulation of silicon oil distribution on transverse section in vertebral body. (a), (b), and (c) represent plane 2, plane 1, and plane 3, respectively. (1), (2), and (3) represent 1 s, 4 s, and 7 s after injection.

**Figure 3:**
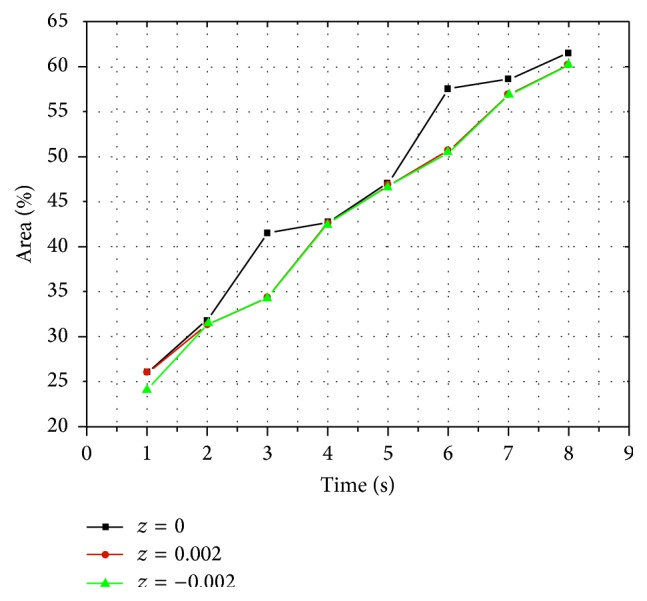
Line chart of silicon oil percentage of distribution area and time on transverse sections in vertebral body.

With gradual injection in vertebral body, as shown in Figure 2, silicon oil diffused into the surrounding area centered by the injection point and formed a circle-like shape on plane 1. Meanwhile, on both plane 2 and plane 3, silicon oil flowed into the surrounding area forming a circle-like shape centered by the intersection points of vertical axis to the transverse section through the injection point and these two planes. The volume concentration of silicon oil decreased gradually from the center to the surrounding area at each time point and on each transverse section (Figure 2). At each time point, there was similar distribution area percent of silicon oil between plane 2 and plane 3, while there was the largest distribution area on plane 1 compared with the other two planes at most time points (Figure 3). The distribution area of silicon oil on plane 1 was similar to those on both plane 2 and plane 3 at few time points (Figure 3).

### 3.2. Silicon Oil Distribution on Longitudinal Sections in Vertebral Body

**Figure 4:**
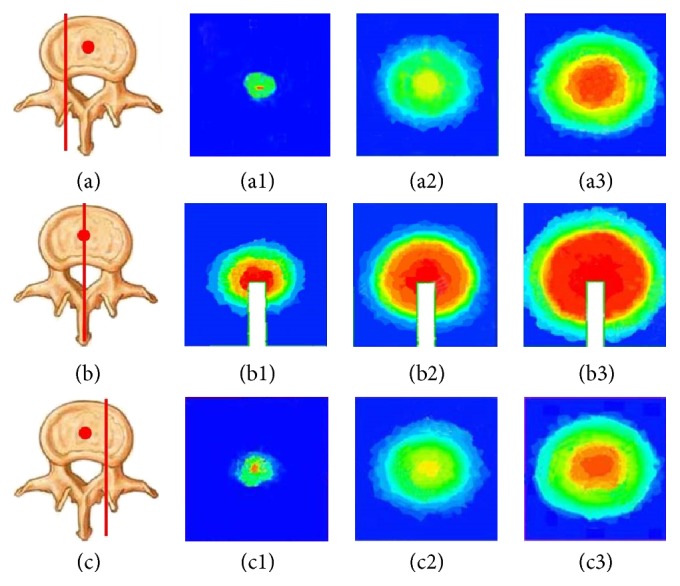
Simulation of silicon oil distribution on longitudinal sections in vertebral body. (a), (b), and (c) represent plane 7, plane 6, and plane 5, respectively. (1), (2), and (3) represent 1 s, 4 s, and 7 s after injection.

**Figure 5:**
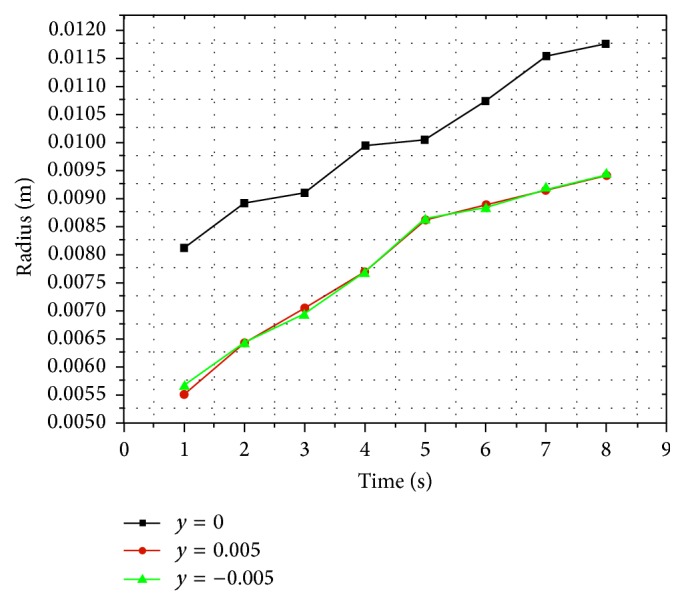
Line chart of silicon oil distribution radius and time on longitudinal sections in vertebral body.

With the gradual injection in vertebral body, as shown in Figure 4, the silicon oil diffused into the surrounding area centered by the injection point and formed a circle-like shape on plane 6. Meanwhile, on both plane 7 and plane 5, silicon oil flowed into the surrounding area forming a circle-like shape centered by the intersection points of vertical axis to the longitudinal section through the injection point and these two planes. The volume concentration of silicon oil decreased gradually from the center to the surrounding area at each time point and on each longitudinal section (Figure 4). At each time point, there was similar distribution radius of silicon oil between both plane 5 and plane 7, while there was the largest distribution radius on plane 6 compared with the other two planes (Figure 5).

Based on the distribution of silicone oil on both transverse and longitudinal sections, the silicone oil flowed into a circle-like shape on plane and eventually formed a sphere-like shape as a whole in vertebral body.

### 3.3. Silicon Oil Distribution on Transverse Sections in Pedicle

**Figure 6:**
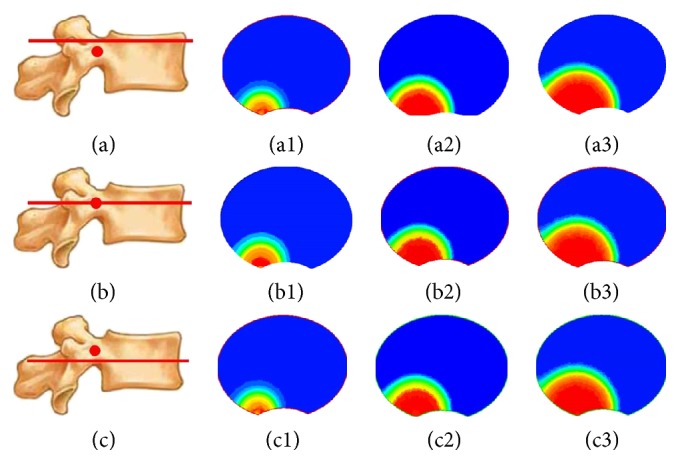
Simulation of silicon oil distribution on horizontal sections in pedicle. (a), (b), and (c) represent plane 2, plane 1, and plane 3, respectively. (1), (2), and (3) represent 1 s, 4 s, and 7 s after injection.

**Figure 7:**
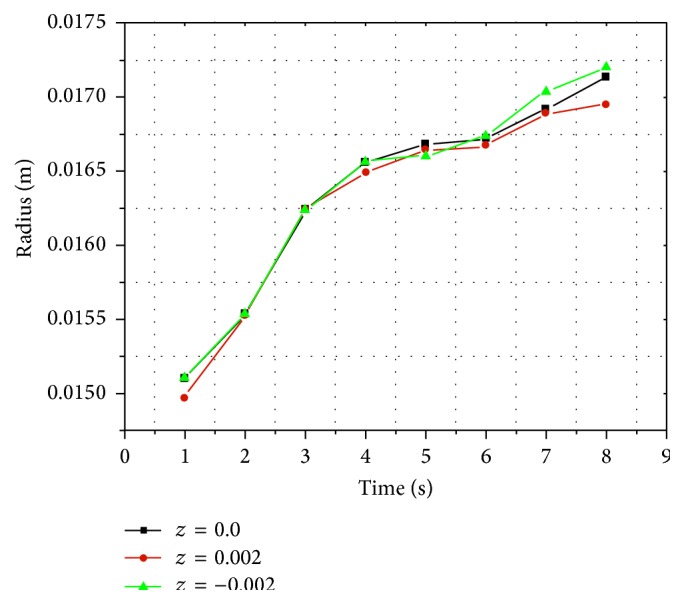
Line chart of silicon oil distribution radius and time on transverse sections in pedicle.

With gradual injection in pedicle, as shown in Figure 6, silicon oil flowed into the surrounding area centered by the injection point and diffused peripherad along the wall after reaching the vertebral wall, eventually forming a circle-like shape. Meanwhile, on both plane 2 and plane 3, silicon oil flowed into the surrounding area and diffused peripherad along the wall after reaching the vertebral wall forming a circle-like shape centered by the intersection points of vertical axis to the transverse section through the injection point and these two planes. The volume concentration of silicon oil decreased gradually from the center to the surrounding area at each time point and on each transverse section (Figure 6). There were no significant differences on silicon oil distribution radius among the three planes at most time points (Figure 7).

### 3.4. Silicon Oil Distribution on the Upper Endplate

**Figure 8:**
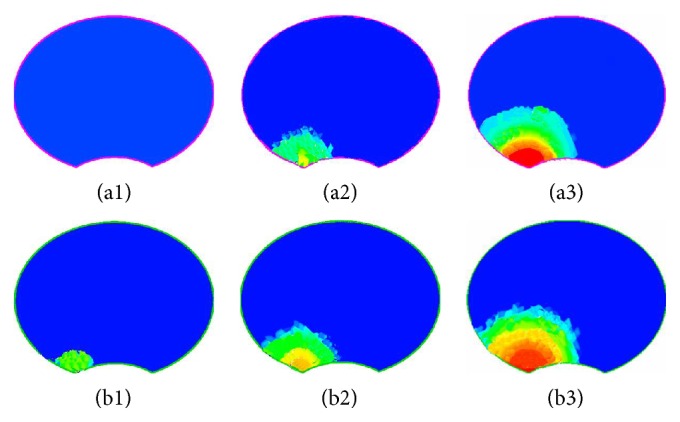
Silicon oil distribution on the upper endplate and plane 4. (a) and (b) represent the upper endplate and plane 4, respectively. (1), (2), and (3) represent 2 s, 4 s, and 7 s after injection.

**Figure 9:**
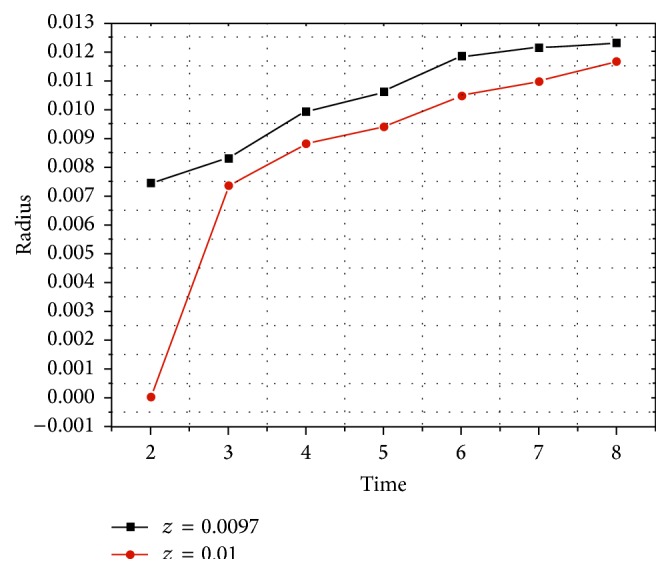
Line chart of silicon oil distribution radius and time on the upper endplate and plane 4.

With the gradual injection, as shown in Figure 8, it was found that appearance of silicon oil was earlier on plane 4 than that on upper endplate. It flowed into the surrounding area and diffused peripherad along the wall after reaching the vertebral wall forming a circle-like shape centered by the intersection points of vertical axis to the transverse section through the injection point and plane 4. The volume concentration of silicon oil decreased gradually from the center to the surrounding area at each time point on both upper endplate and plane 4. Distribution pattern of silicon oil on the upper endplate is smaller than that on plane 4 (Figure 8). At all time points, silicon oil distribution radius on upper endplate was less than that on plane 4 (Figure 9).

### 3.5. Silicon Oil Distribution on the Lateral and Posterior Walls

**Figure 10:**
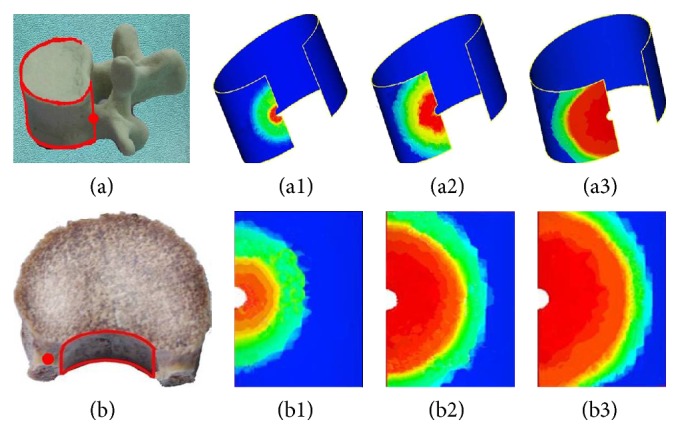
Silicon oil distribution on the lateral and posterior walls. Red points represent injection point; (a) and (b) represent distributions on the lateral and posterior walls, respectively. (1), (2), and (3) represent 1 s, 4 s, and 7 s after injection.

Silicon oil was found on both lateral and posterior walls soon after injection. As shown in Figure 10, it flowed into the surrounding area centered by the injection point as center and diffused peripherad along the wall after reaching the vertebral wall, eventually forming a circle-like shape. The volume concentration of silicon oil decreased gradually from the center to the surrounding area at each time point (Figure 10).

Based on the distribution of silicone oil on transverse sections, upper endplate, and lateral and posterior walls, the silicone oil flowed into a circle-like shape and eventually formed a sphere-like shape as a whole in pedicle.

## 4. Discussion

Currently, PVP is the main surgical treatment for osteoporotic vertebral compression fractures. Bone cement leakage in adjacent venous system or spinal cord causing nerve root injury and spinal cord compression is the most frequent intraoperative complication [11], which occurs at a rate as high as 65% [5]. Baroud et al. [10, 12] studied the transverse and longitudinal permeability of PMMA infiltrating in human vertebrae and relationship between permeability of PMMA and bone mineral density. They also described the relationship between PMMA infiltration rate within vertebrae and time. Tschirhart et al. [13] analyzed the spinal biomechanical strength and stability augmented with different distribution patterns and different volume of PMMA in percutaneous vertebroplasty through a three-dimensional parametric finite element model. However, no research has yet been performed to study the flow characteristics and distribution patterns of PMMA within the vertebrae after injection, especially using computational simulation and analysis.

PMMA is a nonlinear viscous fluid. Its viscosity is unstable which makes it difficult to analyze its flow characteristics and distribution patterns through the fluid mechanics methods [12, 14, 15]. Therefore, this study mainly focused on fluid distribution ignoring the thermal transmission. Moreover, the flow characteristics and distribution patterns of fluid are mainly influenced by poriness, porosity, permeability, and fluid viscosity, so we chose silicon oil with a viscosity of 100,000 cSt matching that of PMMA for study [16–18]. Computational models were established to simulate the flow characteristics and distribution patterns of silicon oil in vertebral bodies. Since the structure of appendix of vertebra is complicated, this study mainly focused on the modeling of vertebral body (appendix of vertebra excluded) by computer.

Silicon oil flow was regarded as continuous medium in this study. In the real process of injection, a large amount of silicon oil was injected in a very short time making the compression and resistance of silicon oil different at the different injection site and injection time. The flow whose parameters change with time is called unsteady flow. So the flow of silicon oil in this study is regarded as an unsteady process. For this reason, the flow characteristics and distribution patterns of silicon oil can be better described using an unsteady model. Because of the air occupying the dry vertebrae, the silicon oil flow inside vertebrae became a two-phase flow between silicon oil and air. There was no chemical reaction and thermal transmission between these two objects, so chemical equations and energy equations were ignored during the computational modeling. Furthermore, we controlled the flow speed of silicon oil into vertebral body by managing the injection pressure. Due to the high viscosity and low flow speed of silicon oil, the flow of silicon oil inside the vertebrae can be regarded as a three-dimensional laminar flow. Therefore, the unsteady incompressible viscous laminar flow in porous medium was used as flow model in the present study [10, 19–21].

Based on the complexity of porous medium, we made the following hypotheses during modeling: (1) the internal framework of vertebral bodies is incompressible and immovable; (2) physical property parameters of solid, liquid, and gas phases are constants, and the thermal transmission, chemical reactions, and phase change among three phases are not considered; (3) basic flow characteristics are controlled by main items of the basic equation; (4) permeability differences between transverse and longitudinal directions are neglected, and cancellous bone within a vertebral body was considered as isotropic rigid material; (5) no slipping occurred near the solid surface (cortical bone); (6) due to the flow characteristics and boundary conditions, Darcy Law $\left(\nabla P=-\left(\mu /\alpha \right)\vec{v}\right)$ was chosen as the constitutive equation in the transfer process of porous medium [19, 22].

Fluent (V6.3, ANSYS) software was used to perform numerical simulation of the flow process. The boundary conditions and initial conditions should be determined firstly. Boundary conditions included the following: vertebral body was considered as elliptical cylinder composed of surface layer with cortical bone and inner space filled with cancellous bone. Because of the very small porosity of cortical bone, the permeability of cortical bone was considered as 0. The vertebral body was simplified to the model with a solid surface and porous medium inside. Initial conditions were as follows: before the injection, porous medium within the vertebral bodies was filled with air (i.e., volume fraction was 1) and no silicon oil existed in porous medium (i.e., fluid volume fraction was 0). Fluid flow was described through methods of mathematical physics, and the motion state and evolution of fluid were described by a group of continuous functions and then analyzed through mathematical methods. We used Euler's methods to change the physical parameters of fluid on different spaces and at different time points into the functions of fixed coordinate and time. Streamlines were then used to transfer the flowing mathematical descriptions into flowing intuitional images [9, 23, 24].

Because of no significant differences on permeability and diffusibility between transverse and longitudinal directions, medium inside the vertebral body was considered as isotropic material [25–27]. Although silicon oil has a relatively high viscosity, it can still be regarded as continuous medium according to the basic principles and basic hypothesis of fluid mechanics. When continuous medium flows in the isotropic material, flow characteristics are similar to each other on different spaces and at different time points. According to the numerical simulation results, the pores within vertebral models were occupied by air before the injection. In other words, silicon oil volume fraction in vertebral body was 0 (shown as blue color in Figures 2, 4, 6, 8, and 10). After the injection, silicon oil started to take up the space originally occupied by air and the air was pushed by silicon oil to flow towards all directions. In volume fraction in the center (shown as red color) approach 1, silicon oil began to spread with the surrounding volume fraction decreasing gradually. The results showed a similar circle-like distribution pattern on all sections within the vertebral body. At the same time point, distribution patterns are similar between those on the two symmetric sections paralleled with the transvers section or longitudinal section through injection point. Silicon oil distribution area was the largest on the longitudinal sections through the injection point at all time points comparing with other longitudinal sections. Though distribution area of silicon oil on the transverse section through the injection point was similar to those on the adjacent transverse sections at few time points (Figure 4), it was the largest on the transverse section through injection point compared with other transverse sections at most time points. This might be caused by overclosure of the selected transverse sections which was within the acceptable scope of engineering calculation error, but it also revealed these flow characteristics and distribution patterns in vertebral body. The results showed that silicon oil flowed towards all directions resulting in a circle-like shape centered by the injection points on each section, eventually forming a sphere-like shape as a whole in vertebral body.

In the pedicle, silicon oil flowed forming a circle-like shape centered by the injection points as center on the transverse sections and spread along the wall when it reached the wall. At the same time points, diffusion radius of silicon oil was similar among the two symmetric transverse sections without injection point and the transverse sections through the injection point. This might be caused by overclosure of the selected sections or the anatomical features and space limitations of vertebral pedicles, but it still reflected these distribution patterns in pedicle. Because of the high viscosity of silicon oil, gravity influence can be ignored within a short period of time after injection. Therefore, we chose upper endplate as study object and selected a close transverse section (plane 4) for comparative study during the simulation of silicon oil flow and distribution in pedicle.

When silicon oil reached plane 4, it diffused faster on the transverse plane than on the longitudinal section. This was the reason for the fact that silicon oil was found on plane 4 earlier than on the endplate. So, the distribution radius of silicon oil was obviously different between plane 4 and upper endplate at the first time point after injection (Figure 9). With the increasing amount of injection, silicon oil diffused into the upper endplate. The concentration gradient became the main power of oil flow when silicon oil reached the upper endplate. Therefore, the distribution radius on the upper endplate became approximated to that on plane 4 as time went on. Observation of silicon oil distributions revealed that flow and distribution of silicon oil were found on the lateral and posterior wall of vertebral pedicles as soon as injection was started. It diffused towards all directions and spread along the lateral and posterior wall after reaching the wall, finally forming a circle-like shape. The volume concentration decreased from the center to the surrounding area at each time point. It was demonstrated that silicon oil flowed resulting in a circle-like shape centered by the injection point on all sections in pedicle and spread along the lateral and posterior walls also forming a circle-like shape. It finally formed a sphere-like structure as a whole.

However, there are several limitations in this study. The specimens in our study were degreased which is different from vertebrae with blood, fat tissue, and bone marrow during the operation. Considering the liquidity of blood and permeability of nutrient foramen, a further accurate vertebral model should be constructed to perform a quantitative study on flow characteristics and distribution patterns of PMMA within vertebral body, which will provide more sufficient theoretical basis and technical support to reduce bone cement leakage risk in PVP.

## 5. Conclusions

Silicone oil flowed and diffused resulting in a circle-like shape centered by the injection point on all sections and it finally formed a sphere-like shape as a whole in vertebral body. It flowed forming a circle-like shape centered by the injection on all sections and spread along the lateral and posterior walls, eventually forming a sphere-like shape as a whole in pedicle. We believe the computational model we constructed is capable of simulating silicon oil flow and distribution in vertebral body. These flow characteristics and distribution patterns can be used to simulate the distribution of PMMA within vertebral bodies, which may provide theoretical basis to reduce cement leakage risk in PVP.
